# DDCNN-F: double decker convolutional neural network 'F' feature fusion as a medical image classification framework

**DOI:** 10.1038/s41598-023-49721-x

**Published:** 2024-01-05

**Authors:** Nirmala Veeramani, Premaladha Jayaraman, Raghunathan Krishankumar, Kattur Soundarapandian Ravichandran, Amir H. Gandomi

**Affiliations:** 1 School of Computing, SASTRA Deemed to Be University, Thanjavur, India; 2 Information Technology Systems and Analytics Area, Indian Institute of Management Bodh Gaya, Bodh Gaya, Bihar, 824234 India; 3 Department of Mathematics, Amrita School of Physical Sciences, Amrita Vishwa Vidyapeetham, Coimbatore, India; 4 Faculty of Engineering and Information Technology, University of Technology Sydney, Ultimo, NSW Australia; 5 University Research and Innovation Center (EKIK), Obuda University, Buddapest, Hungary

**Keywords:** Mathematics and computing, Computational science, Medical research

## Abstract

Melanoma is a severe skin cancer that involves abnormal cell development. This study aims to provide a new feature fusion framework for melanoma classification that includes a ***novel ‘F’ Flag feature*** for early detection. This novel ‘F’ indicator efficiently distinguishes benign skin lesions from malignant ones known as melanoma. The article proposes an architecture that is built in a Double Decker Convolutional Neural Network called DDCNN future fusion. The network's deck one, known as a Convolutional Neural Network (CNN), finds difficult-to-classify hairy images using a confidence factor termed the intra-class variance score. These hirsute image samples are combined to form a Baseline Separated Channel (BSC). By eliminating hair and using data augmentation techniques, the BSC is ready for analysis. The network's second deck trains the pre-processed BSC and generates bottleneck features. The bottleneck features are merged with features generated from the ABCDE clinical bio indicators to promote classification accuracy. Different types of classifiers are fed to the resulting hybrid fused features with the novel 'F' Flag feature. The proposed system was trained using the ISIC 2019 and ISIC 2020 datasets to assess its performance. The empirical findings expose that the DDCNN feature fusion strategy for exposing malignant melanoma achieved a specificity of 98.4%, accuracy of 93.75%, precision of 98.56%, and Area Under Curve (AUC) value of 0.98. This study proposes a novel approach that can accurately identify and diagnose fatal skin cancer and outperform other state-of-the-art techniques, which is attributed to the DDCNN ‘F’ Feature fusion framework. Also, this research ascertained improvements in several classifiers when utilising the ‘F’ indicator, resulting in the highest specificity of + 7.34%.

## Introduction

Skin cancer is caused by abnormal cells and can metastasise quickly if not detected early^1^. Of the number of studies on melanoma using different analytical platforms, one of the pioneering efforts is Computer-Aided Diagnosis (CAD)^2^. These research efforts used automated algorithms to identify cutaneous lesions upfront by analysing image colour, shape, and structure^3,4^. Image segmentation is still a prominent field of study—the progression of segmentation by statistical analysis of medical images^5^. Extraction of characteristics from the lesion region assists in categorisation, although developing such systems has been challenging and sluggish^6^. The majority of automated detection systems use the Asymmetry, Border, Color, Diameter, and Evolution (ABCDE) rule. In 2001, Senan et al. were the first to work with the four aspects of this technique, which involved 122 parameters, and used the conventional models with an automation system employed^7^. Squamous-cell carcinoma (SCC), basal-cell carcinoma (BCC), and melanoma are the three kinds of skin cancer^8^; the former two are less prevalent types and are grouped as non-melanoma skin cancer. While BBC develops slowly, possibly causing tissue damage, it seldom causes mortality and is usually seen in the nasal cavity^9,10^. Squamous-cell skin cancer manifests as a hard mass with a scaly surface and crust, which may lead to the formation of an ulcer^11^. Melanoma is the deadliest and most severe type of skin cancer that can metastasise and become chronic and life-threatening if not diagnosed and treated early. Asymmetry, uneven borders, numerous colours, and distinctive dermoscopic structures are typical melanoma symptoms^12^.

In common, melanoma detection and classification problems have two significant techniques. The first technique uses hand-crafted features, whereas the second employs deep learning approaches, notably Deep Convolutional Neural Networks (Deep CNNs). Deep CNNs have acquired much traction over the past few years for image identification applications^13^. The primary benefit of CNNs is their capacity to retrieve features from datasets autonomously. Thanks to the automated feature extraction method, they may perform categorisation based on the collected features. The most beneficial advantage of using pre-existing CNN architectures is the ability to use transfer learning. These designs have been pre-trained on the Image Net database and may be fine-tuned to meet the needs of our unique model^14^. The CNN models extract convolutional features from Fully Connected Layers (FCN) or dense layers. While these properties generalise effectively to different images, they have difficulties generalising local patterns and dealing with geometric and form differences^15^. Although data augmentation and image-rescaling methods are widely used to circumvent these challenges^16^, they may not always produce the expected effects in many circumstances. For example, randomly cropping photographs may accidentally crop off a Region of Interest (ROI) from the input image, providing a considerable obstacle to performance enhancement.

Our proposed work tackles this issue by creating a segregated dataset that employs many classification algorithms. Comparatively, the BSC dataset is only comprised of difficult-to-classify samples and employs more complex image preparation techniques. These strategies include hair removal treatments that are properly set to ensure minimal data loss. Furthermore, most datasets have an imbalance, with a larger number of benign samples and a smaller proportion of malignant samples. This imbalance exacerbates the difficulties associated with melanoma detection.

The classification framework presented in this article performs the data augmentation techniques to resolve the imbalance in the subsequent dataset while assuring minimum modifications to the images. Hence, it increases the size of the input training set. Another factor to consider when developing a framework is whether to prioritise better sensitivity or specificity. The model's sensitivity evaluates its ability to identify real positives properly, whereas specificity represents its potential to recognise true negatives effectively. Due to the vital relevance of identifying malignant melanoma, this model includes sensitivity and specificity. In traditional melanoma segmentation approaches, methods like thresholding, segmentation, and the ABCDE method are employed. However, when undesired features such as hair or rule markings are present, the performance of these approaches suffers. The ABCDE approach is handy for self-diagnosing melanoma, which evaluates asymmetry, border abnormalities, colour variegations, diameter, and any emerging symptoms or abnormalities in amole of concern^17^. The ABCDE rule is vital in diagnosing malignant skin lesions in the second deck of classification, where the 'F' flag feature is an additional and most influencing bioindicator for image analysis of melanoma.

Despite several works on malignant melanoma, a proper diagnosis from visual inspection remains challenging on account of variables, including poor contrast, illumination, and artefact occlusions with neighbouring tissues. The deprived colour contrast between the targeted skin lesion and the normal skin area makes the segmentation process complex. Furthermore, skin with hair texture, lighting fluctuations, and reflections contribute to the intricacy of dermoscopic images. Because of the variety of skin lesions, reliable identification of malignant melanoma is particularly challenging. Accordingly, this study offers a novel framework consisting of Double Decker Convolution Neural Network (DDCNN) feature fusion with the 'F' flag feature for diagnosing human skin lesion classification to overcome above-mentioned challenges. This system also applies the ABCDEF rule to improve the prediction of melanoma.

The first deck includes a baseline CNN that assists in retrieving difficult-to-classify data using the confidence factor defined by the intra-class variance score. These samples are combined to produce a Baseline Separation Channel (BSC) subjected to hair artefact excision and augmentation. Because complex samples have a more significant number of cases of malignancy than benign instances, data augmentation is used to strike parity between the two classes of input datasets. After the dataset has been enriched and pre-processed, it is put into a CNN to determine bottleneck characteristics. The same dataset is used to extract biomarker details based on the ABCDEF (Asymmetry, Border, Color, Diameter, Evolution and Flag) criteria for the diagnosis of melanoma. These traits, as well as the first deck CNN-extracted attributes, are combined. A system of classification that predicts malignant melanoma is built using the fused feature set. As an aspect of the classification process, gradient-boosting algorithmic classifiers, logistic regression (LR), and perceptron-based multi-layer classifiers are used.

The critical contributions of this presented research work are as follows:

1. A novel Double Decker Convolution Neural Network (DDCNN) feature fusion automated framework is designed for skin lesion classification of malignant melanoma, which integrates medical image indications of skin lesions based on the ABCDE criteria for successful feature selection.
2. Total Dermoscopy Score (TDS) is integrated with the intra-class variance score to address the problem of classifying complex samples occluded with hair artefacts. This score greatly influences the categorisation of challenging skin lesion input images.
3. The ‘F’ flag feature is a new indicator to the binary classification model created exclusively for diagnosing malignant melanoma in skin lesions images, implemented within the DDCNN feature fusion framework.

Experimental findings confirmed the usefulness and efficacy of the suggested DDCNN technique. The performance evaluation demonstrates the model's accuracy in identifying malignant skin cancer and its potential for use in real-world applications. This research offers a new *'*F' flag feature of cutaneous lesions to detect malignant melanoma with DDCNN feature fusion architecture. The suggested approach produced promising results in diagnosing malignant cases by merging dual CNNs with the medical ABCDE rule, adding the intra-class variance score and TDS^18^. The experimental results underline the virtue and efficiency of the proposed method, highlighting its potential for use in practical situations.

## Background

### Feature hand-crafting and its influence on classification

The feature handling in a previous work^19^ was analysed to establish the high-performing predictive accuracy of the different melanoma detection criteria in their investigation. They also assessed the combined precision and recall of these factors in identifying these requirements to determine the existence of melanoma. Another study^20^ was conducted to increase classification performance by random sample mixes of skin textures, such as intensity correction and noise addition with ABCDE features. The feature collection was reduced to two dimensions using Principal Component Analysis (PCA), yielding an excellent area under the ROC plot of 94%.

Another study^21^ offered a mix of hand-crafted attributes, a feature optimisation framework, and K-Nearest Neighbours (KNN) for melanoma diagnosis, aiming to improve the accuracy of the categorisation process. Researchers^22^ have also used an alternative approach, proposing a melanoma detection system with geometrical and texture features. Furthermore, this research^23^ presented an ensemble approach to melanoma categorisation, utilising an autonomous neural network to construct lesion regions using ensemble-based classification.

### Preliminaries of deep learning techniques

Another feature extraction method is deep learning, which uses Convolutional Neural Networks (CNNs) to derive features dynamically without needing expert knowledge. Instead of handmade feature extraction approaches, deep learning learns these features during training compared to hand-crafted feature extraction approaches. CNNs, on the other hand, are highly computationally complex and require massive datasets for training to enable good feature extraction. In addition to CNNs and hand-crafted algorithms, hybrid approaches have lately gained popularity. The "Land Use Identification" (LUI) task^24^ was achieved in the study. The deep CNN model is designed for the classification task from scratch. CNN and transfer learning were used in a CNN-based survey of feature extraction. These attributes were fed into machine learning (ML) systems like Decision Trees, Random Forests, Naive Bayes, Support Vector Machine (SVM), and KNN classifiers.

Several works on melanoma have employed artificial intelligence to extract features from dermoscopic skin cancer of multiple classes. Using the Haar cascade algorithm^25^, the collected characteristics were input into machine learning algorithms on the facial images to recognise the emotions between six classes. In another study, clinical image samples were fed into machine learning algorithms that used the hybrid wavelet transform, employing the entropy and several other clinical metadata to produce adequate findings^26^. Furthermore, dermoscopic images were utilised in a study employing the ISIC dataset to test seven machine learning methods. The study^27^ used a fusion approach that combines Sorted Block Truncation Coding (SBTC) and Gray Level Co-occurrence Matrix (GLCM) characteristics. Machine learning algorithms were applied to detect and diagnose melanoma in a study that combined two versions of BTC feature extraction approaches with different colour spaces^28^. Additionally, study^29^ suggested combining GLCM elements with several colour samples. It put them through their non-distractive process to extract textural information from medical skin input images. The findings showed that this approach allowed images to be classified as benign or malignant. One research article^30^ applied the cosine transform on images and discovered that a blend of decision support tree and random forest classifiers produced better results, providing machine learning algorithms using a fraction coefficient expressing the elements of dermoscopic skin images.

Moreover, many researchers have contributed multisampling learning techniques (MLTs) to discern benign and malignant human skin lesion images^31^. To optimise dermoscopic images^32^, one study introduced practical preprocessing approaches for effective image enhancements that promise the removal of unnecessary occlusions on the ROI—for instance, hair removal and cropping and noise removal techniques. Furthermore, in another research^33^, the Multi-Sample Learning (MIL) approach was used, yielding a specificity of 87.50% in identifying cancerous and dermoscopic nevus images. Another study^34^ focused on cutting-edge advances in melanoma image classification, such as classification approaches that use colour and texture attributes without preprocessing the photos. One work^35^ also provided a set of autonomous learning techniques for the accuracy of the classification step. Herein, this study drew inspiration from the former literary works, aiming to contribute to the development and implementation of CNN for melanoma classification.

### Hand-crafted feature fusion framework and deep learning

Several studies have investigated the combination of handmade feature extraction and deep learning approaches. Using the ResNet-50 and DenseNet-201 CNN architectures, Li et al.^36^ suggested a fusion strategy that merges clinical representation and the Light Gradient Boosting Machine (LightGBM) algorithm with deep learning. This approach identified textural, colour-based, and form attributes with an accuracy of 85.3%. The CAD system for melanoma diagnosis was created^37^, combining carefully crafted characteristics based on the ABCD features derived by ensemble learning. Another study^38^ proposed integrating image pixel features and clinical metadata using a ResNet-50 network.

This proposed study can be distinguished from the reviewed literary works in several aspects. While numerous research studies focused on predicting malignant melanoma, clinical diagnosis using naked-eye inspection of images by experts is difficult due to various reasons, including class imbalance, noise (such as hair occlusions), high feature dimensionality, image size, and resolution discrepancies. The proposed methodology resolves these issues successfully and creates a classification paradigm for prognosticating malignant melanoma. Deep learning combined with hand-crafted feature extraction has shown considerable potential in melanoma identification. Training the sample images with artefacts in a deep-learning model was quite perplexed in existing systems. The proposed framework’s specificity was enhanced according to the experimental results. The primary goal for this study was to effectively overcome the mentioned challenges by incorporating a segmented strategy that utilises intra-class variance score, data, and augmentation, ABCDE with the new 'F' flag rule that will become prevalent in early diagnosis of challenging skin lesions, thereby bringing down the margin of error.

## Proposed methodology

**Figure 1:**
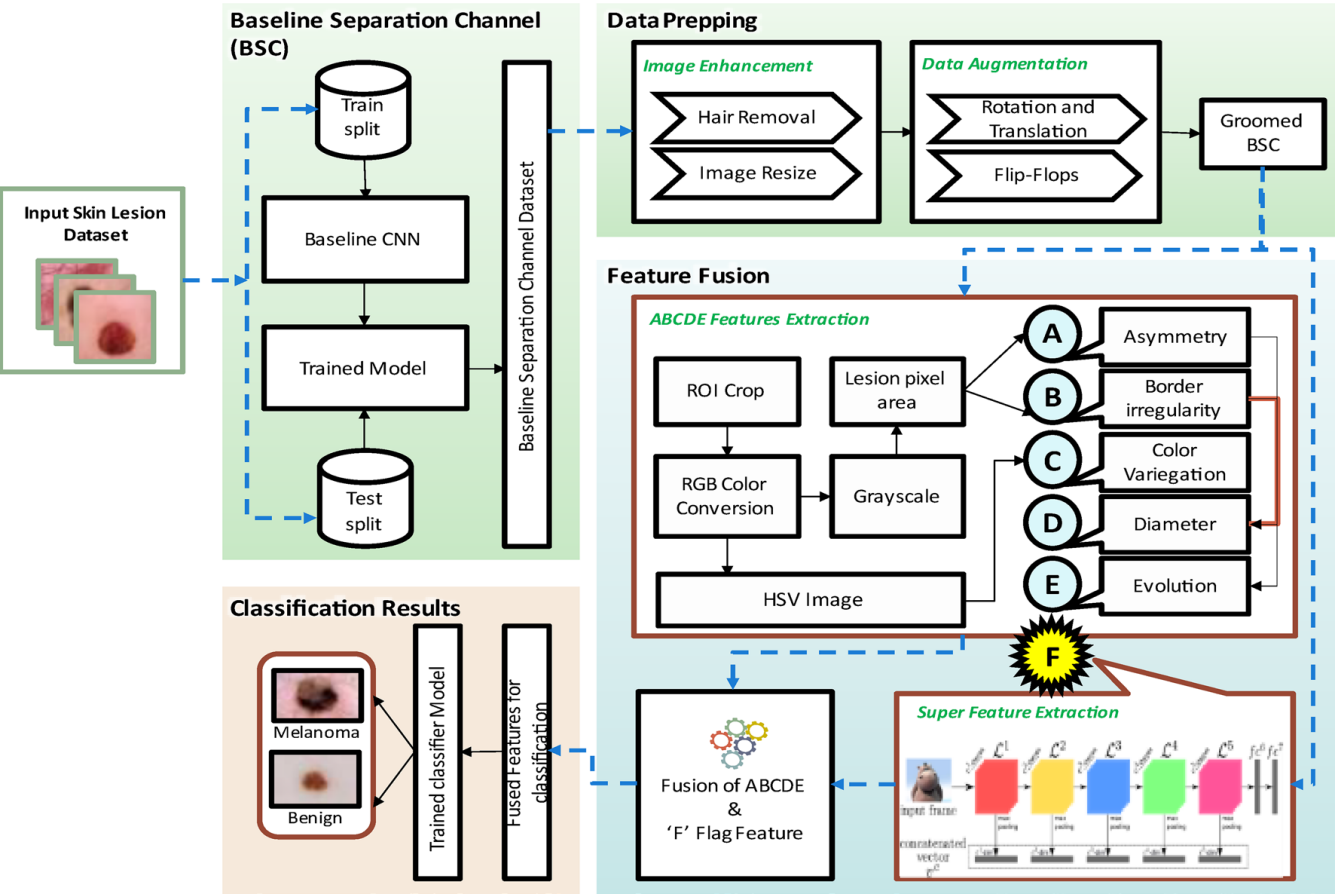
Architectural diagram of the proposed DDCNN system.

The proposed architecture comprises deck one, which contains the baseline CNN, and deck two with CNN for feature extraction and fusion of the ‘F’ flag feature. The classification employing the DDCNN features is the main element of the system. CNNs in the DDCNN system follow the Asymmetry, Border, Colour, Diameter and Evolution (ABCDE) rule to produce a trustworthy and solid classification archetype for exposing malignancy in skin lesions. Algorithm 1 elucidates the sequential workflow of the DDCNN feature fusion framework. Figure 1 exhibits the architectural diagram of the proposed DDCNN system.

**Algorithm 1:**
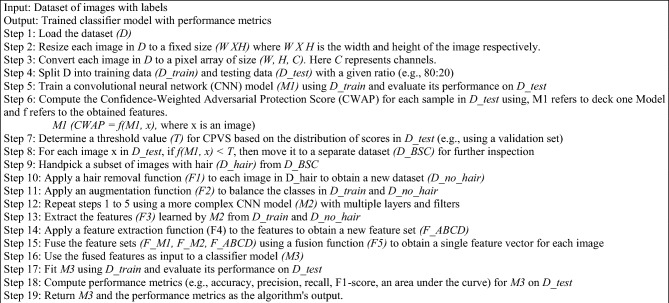
DDCNN () Sequential workflow representation.

### Deck-one baseline CNN

The first deck baseline CNN^39^ typically performs primary classification, recognises problematic samples like those with hair artefacts and processes the data samples in the early steps. The intra-class variance score calculates the probability of benign or malignant lesions. The Baseline Separated Channel dataset (BSC) is a separate dataset that contains the problematic samples, which is vital in the segregation of the dataset. The cut-off point over the intra-class variance score is the confidence factor, established by comparing divergence with the error ratio.

The BSC is subjected to preprocessing techniques to eliminate contaminants like hair to guarantee data balance and cleanliness. A summary of the features used in the CNN model can be found in Table 1. Four convolution blocks comprise the design, each with a unique arrangement of convolutional, normalisation batch, maximum pooling, and dropout layers. Flattened and dense layers with dropouts are placed after these blocks.

**Table 1:** CNN specifications applied in BSC.

Layers	Descriptions	Activations
convolutional2D_1	Size of (3,3)- 15 filters	ReLu
batch_norm_layer1	–	–
convolutional2D_2	Size of (3,3)- 15 filters	ReLu
batch_norm_layer2	–	–
max_pool2D_1	(2,2) pool size	–
dropout_layer1	0.2 Rate	–
convolutional2D_3	Size of (3,3)- 15 filters	ReLu
batch_norm_layer3	–	–
max_pool2d_2	(2,2) pool size	–
dropout_layer2	0.2 rate	–
convolutional2D_4	Size of (3,3)- 15 filters	ReLu
batch_norm_layer4	–	–
max_pool2d_3	Size (2,2)	
flatten_layer1	–	–
dense_layer1	100 units	ReLu
dropout_layer3	0.2Rate	–
dense_2	2 Units	SoftMax

To further explain, consider the example of (F*g) (t), where t is a real numerical variable and *g (*$\tau$*)* represents the convolutional process of *F(t)*. Equation (1) is processed at each convolutional layer to carry out the whole image's convolving procedure:

1 $$\left(F*g\right)\left(t\right)={\int }_{-\infty }^{+\infty }F(\tau ) g\left(t-\tau \right)$$

Overall, the technique requires harrowing sample extraction in the first layer and using a baseline CNN for classification. The intra-class variance score is computed to distinguish between benign and malignant lesions. The samples are divided into the BSC according to the confidence factor. The BSC is pre-processed to remove hairs, and the CNN model, presented in Table 2, operates on the cleaned data using convolutional blocks and the following layers. The most noticeable characteristics (maximum value) inside each pooling zone are chosen to down-sample the feature maps by utilising maximum pooling layers. Here, Eq. (2) calculates the size of a max pooling layer's output feature map:

2 $$\frac{{n}_{fm\_h}-{f}_{s}+1}{{L}_{s}}*\frac{{n}_{fm\_w}-{f}_{s}+1}{{L}_{s}}*{n}_{ch}$$

where ${n}_{fm\_h}$ stands for the input feature map's height;${n}_{fm\_w}$ is its width; *n*_*ch*_ represents the total number of channels;${f}_{s}$ is the filter's size; and ${L}_{s}$ denotes the stride length. ReLU and SoftMax activation layers are used in the architecture. An example of a linear activation function is the Rectified Linear activation function (ReLU), which results in the output value as positive and zero otherwise for the corresponding input. Equation (3) is used to calculate the value provided to the ReLU activation function, represented as *f(Z)*:

**Table 2:** Deck two CNN model of ABCDEF feature extraction.

Layers	Descriptions	Activations
convolutional2D_1	Size of (3,3) -32 filters	ReLu
convolutional2D_2	Size of (3,3)- 64 filters	ReLu
max_pooling2D_1	(2,2) pool size	–
convolutional2D_3	Sze of (3,3)- 128 filters	ReLu
max_pooling2d_2	Pool size (2,2)	–
convolutional2D_4	Size of (3,3)- 256 filters	ReLu
max_pooling2d_3	Pool size (2,2)	
flatten_Layer1	–	–
dense_Layer1	256 units	ReLu
dense_Layer2	128 units	ReLu
dense_Layer3	64 units	ReLu
dense_Layer4	32 units	ReLu
dense_Layer5	2 units	Sigmoid

3 $$f\left(Z\right)=\left\{\begin{array}{c}Z, Z>0\\ 0, Z\le 0\end{array}\right.$$

A scaling function called SoftMax converts the input data into probabilities. The vector representing the probability for each conceivable outcome, corresponding to various classes, is an outcome obtained from the SoftMax activation function. The SoftMax function's denominator has a cumulative term that serves as a normalisation factor for the *i*^*th*^ element *(z*_*i*_ $\in$ *Z)* of the supplied vector *(z)*, guaranteeing that each value of the returned vector is 1. Equation (4) illustrates the mathematical definition of the SoftMax activation function, where *k* stands for the total number of classes:

4 $${e}^{z}i=\frac{{e}^{z}i}{\sum_{j=i}^{k}{e}^{z}i}$$

Binary cross-entropy and the Adam optimiser are the loss functions used in this model. The Adam optimiser is set up during the model's compilation with 0.001 as an initial learning rate and a loss function of binary cross-entropy. Accuracy serves as the evaluation metric. The training set is used with a batch limit of 40 to train the CNN model. Basic preparation operations are performed on the dataset before training, including scaling and array format conversion. The trained CNN framework is then used to generate predictions on the test dataset. The likelihood of each test sample being benign or malignant, as well as the anticipated class and the actual class labels, is computed. The confidence factor is the contradiction between the benign and malignant lookalike samples, also known as the intra-class variance score. For each test sample, this computation is completed. Upon examination, it is found that the error ratio, which is summarised as the proportion of several erroneous samples (where the class that was predicted falls short of the actual one classification) and the total number of samples that lie within that scale, is 0.05 when the confidence factor drops below 0.999995. This shows that, within this range, the model is 95% accurate in its predictions.

Further investigation reveals that the frequency of incorrectly categorised samples rises as the certainty factor falls below 0.99, leading to an error ratio of 0.3, which indicates that the model is accurate only 70% of any given time within this range. Following several iterative trials with different cut-off values, it was discovered that 0.999995 is the threshold value that produces the necessary problematic samples (with a certainty factor of 0.999995). Then, these complex samples are transferred to a separate dataset. Algorithm 2 explains the steps in creating an independent dataset for difficult-to-classify samples.

**Figure:**
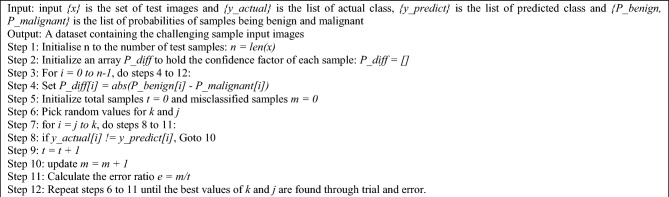
Algorithm 2 Do_BSC() Pseudocode representation of the baseline separated channel.

The misclassification in the deck one is controlled and regularized by the error ratio ‘*e*’ given in Algorithm 2. Given that the allowable 0.05 error ratio was set, the numerical values of *j* and *k* were calculated to be 0.999995 and 1, respectively. This choice is made in light of the algorithm's capacity to classify data accurately 95% of the time. The remaining data points are then moved to a confined dataset if their intra-class variance score is less than 0.999995. The best values of *k* and *j* were evaluated by the image patches that do not find any more hairy pixel in the processed patches. In that case, the image will terminate and go to the final classifier, and the rest is performed as given in the Algorithm 2 sequential steps.

Several image processing techniques are used to efface the hair from the lesions to simplify the categorisation procedure. The image is first changed to grayscale to streamline the ensuing processes. Then, using the hair to represent the black region against the light-coloured backdrop of the lesion, a black hat filter accentuates the bright locations of interest inside the darker background. This filter effectively highlights the hair in the photograph, which is followed by a thresholding approach. The fundamental idea of thresholding is to assign a specific amount to a pixel only if it exceeds a predetermined threshold; otherwise, an alternative value is given. In the resulting image, hair is the only feature that stands out against the dark background, which is handled using Algorithm 3.

**Figure:**
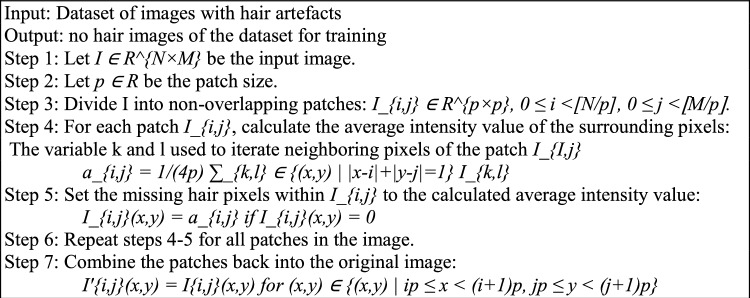
Algorithm 3 Hair_removal() process.

**Figure 2:**
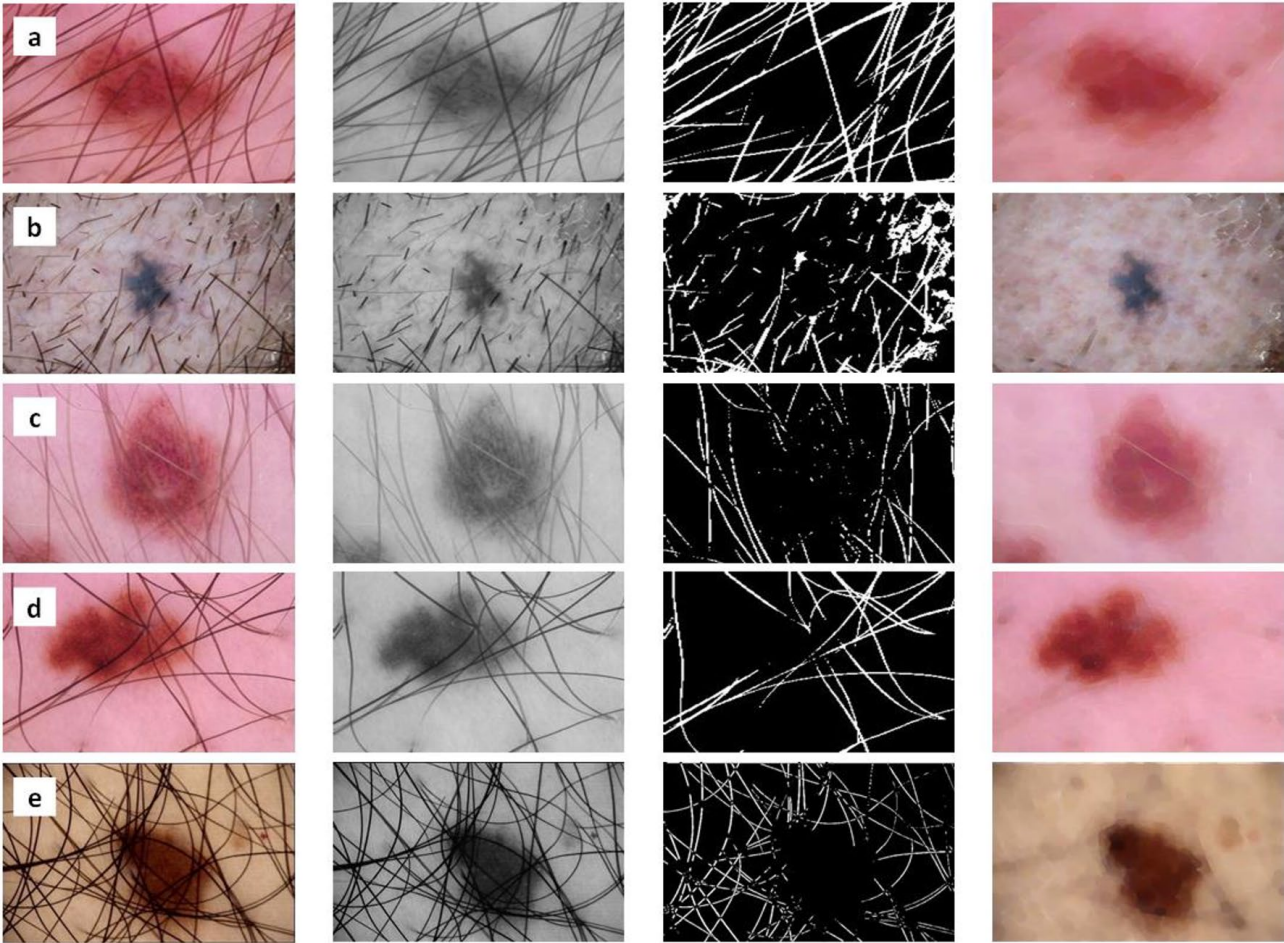
Hair removal process from different sample images of hair from ISIC 2019 and ISIC 2020 datasets.

Finally, this image of highlighted hair, which is from the threshold masked image and the original image with the hair, was subjected to an inpainting algorithm. The parameters x, y refers to the image coordinates that would be used in the hair removal Algorithm 3. The variables k and l are used as iterators for the summation over the neighboring pixels of the patch *I_{i,j}.* These variables are used to traverse the set of neighboring pixel coordinates. Whereas, the *[N/p]* and *[M/p]* notations represent integer division. It denotes the maximum number of patches that can fit in the *N* and *M* dimensions of the input image when the patch size is *p*. It ensures that patches are non-overlapping. In Fig. 2, the original image was modified to remove the masked areas that match the hair, resulting in an image with no visible hair. A lot of effort is taken to prevent adding noise during the inpainting algorithm, so the local neighbourhood around the pixel to be painted must be considered. The normalised weighted sum of all the pixels in the neighbourhood is then calculated to replace the missing pixel. The pixels that are near the target location are given more attention. This method produces an image where the original hair-containing pixels are effectively replaced, resulting in a hair-removed image with no additional pixels or noise.

Several data augmentation approaches are then used to improve the Baseline Separated Channel (BSC) data balance. These consist of horizontal flipping, random noise, and rotation at random angles. In the image augmentation process, a random rotation of 25% towards the left and 25% in the opposite direction is applied to each image, which mainly contributes to adding variety and diversifying the dataset. Mirror images are produced by directly flipping the image array using the horizontal flipping function. The dataset size can be increased, and orientation variants can be included with the help of this augmentation technique. Gaussian-type noise addition is chosen to include randomness in the images. The noise addition is fixed at 0.05 to prevent significant changes in the image. The Gaussian distribution's mean and variance are other parameters left at their default settings. By using these strategies for data augmentation, the BSC dataset is expanded, boosting data balance and diversification to improve training.

### Deck two CNN &feature fusion

The second deck CNN architecture then takes the BSC dataset as its input. Table 2 lists the specifics of the CNN model that contains five dense layers, of which the 64-unit dense layer is chosen for feature engineering. The CNN's dense layers automatically extract the pertinent features and are layered just before the last activation functions. Given its function in activations and classifications, selecting the final dense layer is helpful since it allows for the extraction of precise information. The binary cross-entropy loss function and Adamax optimiser are both used. As mentioned above, these features are applied to each training and testing image and each dataset is stored separately.

### Extraction of ABCDEF features

The ABCD rule^40^ is a method that medical experts use to identify if a mole is benign or cancerous. The abbreviation ABCD stands for the following rules. The letter *A* signifies the word asymmetry, which denotes that the mole's two sides differ. *B* stands for border, emphasising jagged, notched, uneven, or blurry borders. The letter *C* stands for colour, indicating the existence of irregular colouring with different tones of brown or black as well as the potential presence of pink, red, white, or blue colours. The letter *D* represents diameter, which signifies that a mole's diameter is greater than 0.6 cm. The ABCD bio indicator of melanoma has the rule^41^ that includes assessing the common traits of asymmetry of the lesion in images, irregular borders of the target lesion, colour distribution and multiple colour variations, and diameter. Equation (5) presents the Total Dermoscopy Score (TDS), indicating if a mole is benign or malignant, based on the ABCD rule:

5 $$TDS=1.3*A+0.1*B+0.5*C+0.5*D$$

A lesion is considered benign if its TDS is lower than 4.75. The lesion is deemed suspicious when the TDS is between 4.75 and 5.45. A TDS greater than 5.45 implies cancer.

A patient’s mole image is analysed to determine the ABCDE features, and the relevant numerical attributes related to these features are then obtained^42–45^. The method for extracting the ABCDE characteristics is described in Algorithm 4. The image is first put through a process that isolates the mole's primary area of occupancy, removing the surrounding areas.

**Figure:**
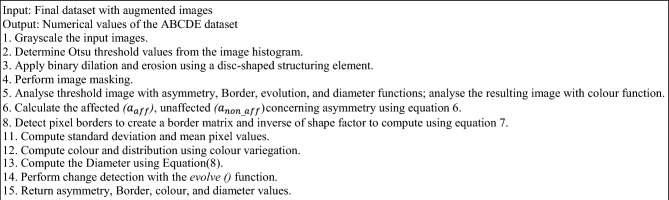
Algorithm 4 Extraction of ABCDE features.

This separated image is then saved independently and subjected to additional grayscale processing to simplify recognising asymmetry, border, and diameter. The RGB values are transformed into HSV digits using the mole's original colour image. The Otsu threshold technique^46^, which repeatedly chooses the best threshold value to separate foreground and background pixels, is simultaneously applied to a black-and-white image.

Following that, the output is exposed to the process of binary dilation performed by the 3 × 3 structuring element, which sets the output value as the highest among the nearby pixels. The image is then subjected to binary erosion to determine the output pixel's value as being the lowest among the neighbouring pixels. Each of these edited photos is kept separately.

The image is then masked, allowing for the selective covering up or revealing of specific areas. A separate file is also kept for this masked image. The asymmetricity, border irregularity, colour variegation, and diameter attributes are taken from the two processed images.

Equation (6) is used to determine the impacted and unaffected areas separately to obtain the asymmetry parameter A:

6 $$A=\left(\frac{\left({a}_{aff}/{a}_{non\_aff}\right)*100}{10}\right)$$

where ${a}_{non\_aff}$ is the unaffected area; and ${a}_{aff}$ is the affected area.

Concerning the border parameter, Eq. (7) yields the lesion's border perimeter:

7 $$B=\left(\frac{(p*p)/(4*3.14*{a}_{aff})}{10}\right)$$

where *p* is the lesion perimeter; and ${a}_{aff}$ is the affected area. The RGB colours are converted to HSV values to compute the volatility for the colour parameter. The colour attribute is obtained by dividing the resultant standard deviation by 10.

**Figure:**
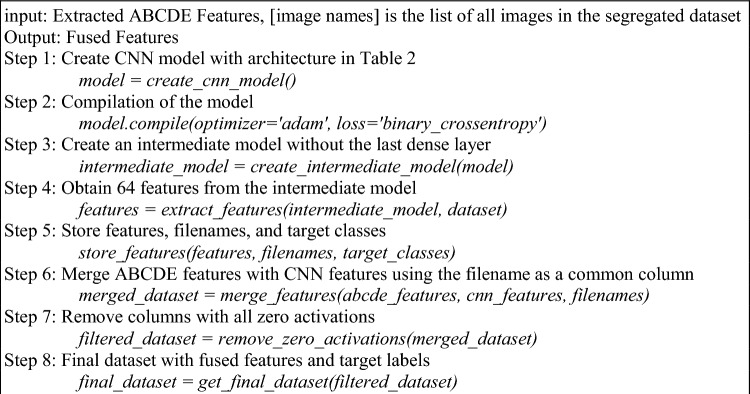
Algorithm 5 Feature fusion framework.

**Figure 3:**
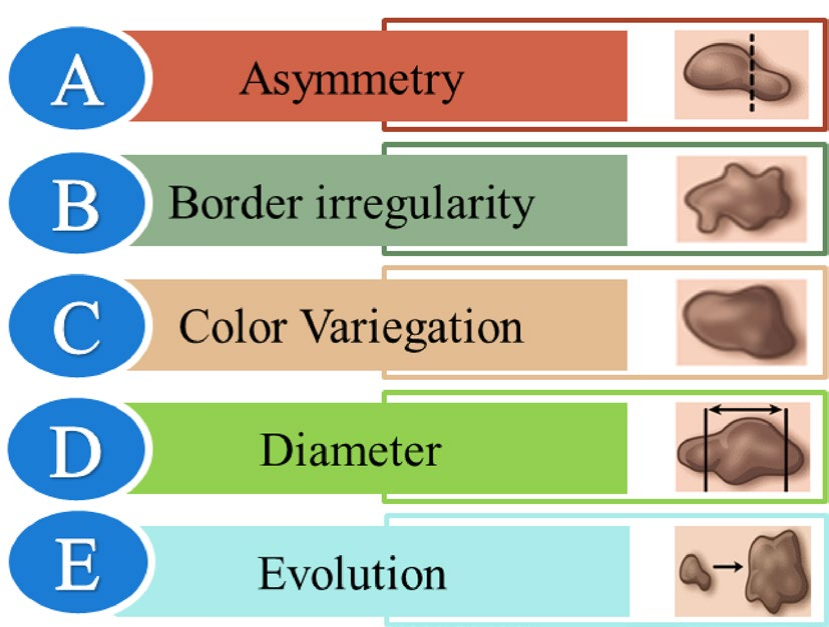
Extraction of Asymmetry, Border, Colour, Diameter and Evolution characteristics.

The impacted region and its perimeter are used to calculate the diameter parameter via Eq. (8):

8 $$D=\sqrt{(4*a)/(p*10)}$$

where ${a}_{aff}$ stands for the affected area; and *p* is the perimeter. Within a segregated dataset, these metrics have been calculated for each sample. A CNN approach also yields measures for asymmetry, border, colour, and diameter in addition to crucial information. A variety of machine-learning approaches use a combination of these metrics and data. Figure 3 provides a visual depiction of the overall process of this research module. The evolution of the skin lesion is calculated by observing the changes in the area and border features of the lesion over time intervals. In the proposed work, it is considered a less significant feature since the implementation focuses on early detection of melanocytic lesions. Algorithm 5 describes the procedures involved in merging these various aspects. The ABCDE features that are extracted is fused with this novel ‘F’ flag feature to add features that influence early diagnosis based on presence and absence of a blue-white veil (BWV). The feature fusion process was deduced as a sequential algorithm where the final classification of the cancerous vs. non-cancerous skin lesion are distinguished as two targets of melanoma and non-melanoma respectively.

### ‘F’ Flag feature

This study's 'F' feature fusion involves fusing the new bio-indicator to the other ABCDE feature for melanoma classification to distinguish between cancerous and non-cancerous lesions. This 'F' flag feature discriminates the classes based on the presence and absence of a specific characteristic called a blue-white veil (BWV), which is a numerical feature extracted from the images through pixel-level processing. To characterise the *'*F' flag feature and impose a higher prediction rate, it is also blended with the texture information of the skin lesion. The confidence factor is used for the induced decision value for the decision support hierarchical tree. This feature supported as a flag is considered one of the prevalent features and is defined in Eq. (9):

9 $${S}_{Flag1}=\frac{Area\, of \,the\, BWV}{Area\, of\, the\, Skin\, Lesion}$$

Herein, the pixel-level features were obtained from the samples during the classifiers training phase. Among all features, the most contributing two specific properties, notably *F7* (the pigment of grey-blue areas) and *F13* (atypical globules pigment network), are necessary from the pixels during the rule application phase. The decision tree uses these two properties to make categorisation decisions. Following the application of the decision tree to an image, the applied rules are used to build the initial binary veil mask. A 3 × 3 majority filter is used to enhance the smoothness of the mask boundaries^47^. The majority class label in each pixel's 3 × 3 neighbourhood is substituted for its value throughout this filtering operation.

The problem with using ${S}_{Flag1}$ alone is that a blue nevus might be misclassified as melanoma due to its high percentage of blue-white areas. The misclassification of skin lesions based on color spots is solved by these geometrical characteristics which is expressed as the lesion's ellipticity and/or circularity^48^: The circularity of a lesion can be characterised as given in Eq. (10):

10 $${S}_{Flag2}=\frac{\frac{1}{px}\sum_{n=1}^{px}|\left|\left({r}_{n}-{c}_{n}\right)-\left(\overline{r },\overline{c }\right)\right||}{{\left({\left(\frac{1}{px}\sum_{n=1}^{px}|\left|\left({r}_{n}-{c}_{n}\right)-\left(\overline{r },\overline{c }\right)\right||-{m}_{R}\right)}^{2}\right)}^{1/2}}$$

where *px* is the number of points on the lesion boundary; $\left({r}_{n},{c}_{n}\right)$ is the spatial coordinate of the ${k}^{th}$ boundary point; and *(r̄, c̄)* is the centroid obtained from the skin lesion image. The third flag feature and measure of the ellipticity of a lesion is given in Eqs. (11) and (12) respectively:

11 $$S_{{Flag3}} = \left\{ {\begin{array}{*{20}c} {16\pi ^{2} M,} & {if\,M \le \frac{1}{{16\pi ^{2} }}} \\ {\frac{1}{{16\pi ^{2} M}},} & {Otherwise} \\ \end{array} } \right.$$

12 $$M=\frac{{\mu }_{30}{\mu }_{03}-{{\mu }^{2}}_{11}}{{{\mu }^{4}}_{04}},$$

where *M* is the moment invariant; and ${\mu }_{xy}$ represents central moments. The inclusion of ${S}_{Flag2}$ and ${S}_{Flag3}$ is justified by the possibility that extremely circular and/or elliptical lesions with flag patches represented as the new bio-indicator 'F' can be separated from melanomas. ABCDEF combined the method with fivefold cross-validation to create a classification model.

### Classification with DDCNN feature fusion framework

This study employs seven distinct classifiers that can be classified into three groups for effective skin lesion classification of dermoscopy images. These categories are deep neural network classifiers, ensemble-based classifiers, and long-established algorithmic classifiers.

In this work, a multi-layer perceptron was used as the deep learning classifier, and the combination of the gradient boosting classification algorithm, XG boosting classifier, and bagged classifier constitute the ensemble learning classifiers. Decision trees, SVM, and logistic regression were applied as the machine learning classifiers. Recognising each classifier's distinctive qualities and the requirement to choose the best classifier for this particular use case are the justifications for using multiple classifiers. Out of several experiments, selected the best option by testing these different classifiers. Bagging is preferred because of its ignoble variance; XGBoost was considered because of its speed; and SVM was chosen due to its potential with smaller datasets. Each classifier has unique advantages, and thus, the second deck CNN has the ensemble classifier that trained the model to be highly exact in performing the classification task of distinguishing malignant samples from typical lesions.

### Deep neural learning classifier

Through artificial neural networks, deep neural networking is an aspect of machine learning that imitates human experts regarding image processing capacity. The multi-layer perceptron (MLP) is a prominent deep learning classifier for issues related to classification.

### Multi-layer perceptron

MLP was chosen because it works well with numerical datasets and has a remarkable knack for spotting patterns and trends. Each node in an MLP functions as a neuron and is divided into an input layer, hidden layers, and an output layer. Neurons, besides the input nodes, use nonlinear activation functions. The MLP uses back propagation, a training method, to improve the neural network's performance. In Table 3, the model's architecture is displayed.

**Table 3:** MLP specification parameters.

Layers	Output Shape	Parameters
Input	(None, 784)	0
Dense_1	(None, 512)	4,01,920
Dense_2	(None, 256)	1,31,328
Dense_3	(None, 2)	514
Activation	(None, 2)	0

In this work, an MLP structure was constructed with four layers, including a sole input layer, a trio of hidden layers, each with 64 numbers of nodes, and one output layer. The activation function was performed by the Rectified Linear Unit (ReLu).

### Ensemble classifiers

Ensemble classifiers are a group of classifiers that integrate their separate judgements, frequently by voting, to categorise brand new data. This method improves classification robustness and accuracy^49^.

### Functional gradient boosting classifier

Due to its excellent accuracy and effectiveness in two-class classification situations, the gradient-boosting classifier was chosen. Each model in gradient boosting seeks to outperform its forerunner by lowering errors. It concentrates on blending an entirely novel model to the residual errors produced by the preceding model rather than fitting a model to the complete dataset at each stage.

Three essential elements are involved in gradient boosting. First, it is crucial to optimise the loss function, which measures the model's accuracy in making predictions based on the available data. Second, predictions are made using weak learners like decision trees. These decision trees find the ideal division points based on metrics like the Gini index.

Eventually, the third element of this method enables the supplemental inclusion of numerous learners, even if weak, to reduce the loss of function. Each tree has been included using gradient descent to minimise the loss. Twenty estimators in total were used in this study, and the best learning rate for the classification was determined by testing several learning rates using the deviance loss function.

### Bootstrap aggregating classifier

The bagging classifier was chosen since it reduces variance and guards against overfitting while combining numerous weak learners to produce a robust classifier. This kind of classifier aggregates the predicted outcomes of each subset of the original data to achieve the final prediction.

The bagging classifier slashes the variance of computations like decision trees by including randomisation early in the process and using an ensemble. In this work, a Decision Tree Classifier was employed as the base classifier, and 10 base estimators were used. A distinct portion of the training data, chosen randomly with replacement, was used for each decision tree's training. Each decision tree's output was combined to create the final categorisation during testing. By using a voting mechanism amongst the base classifiers, this method successfully lowered overfitting and variance.

### eXtreme gradient boosting(XGBoost) classifier

A machine learning approach called XGBoost uses gradient boosting to merge different models. Due to its exceptional versatility and quickness, which make it the perfect option for datasets of minimal to intermediate sizes circumstance, also chosen the XGBoost classifier. Parallel processing^50^ is used in its entirety of our proposed work. Boosting teaches models in a sequential process, where each new model seeks to correct the faults caused by its predecessors instead of training each model separately. An essential benefit of this iterative technique is that the freshly developed models concentrate on fixing the mistakes introduced by the earlier estimators. The method uses CART-based decision trees, and for this specific model, 100 estimators were used as it is known that this method can overfit. To address this problem, added this eta parameter to prevent overfitting. The technique includes several improvements, such as cross-validation, sparsely awareness (adding samples encountered infrequently), and regularisation (using L1 and L2 regularisation to avoid overfitting).

### Machine learning classifiers

Making predictions about the class to which a set of supplied data points belongs is the challenge of ML classifiers. These classes are frequently referred to as labels or targets.

### Decision support trees

The decision trees were designated in this work due to their effectiveness in handling numerical data and ease of use. Additionally, they provide speedy and effective processing and are simple to visualise^51^. Decision trees utilise both the CART and index of Gini methods to locate the possible inferior sub-tree that precisely reflects the input data^52^. The feature with the biggest information gain, as determined by the value of the entropy Eq. (13), has been selected as the decision tree's root node:

13 $$Entropy\left(x\right)= \sum (p\left(x\_k\right)*{log}_{2}p\left(x=k\right))$$

where information gain is given in Eq. (14):

14 $${I}_{g}\left(feature\right)=Entropy\left(data\right)-Entropy\left(feature\right)$$

The attribute with the lowest IG is split by applying the Gini Index to the attributes of the CART decision-tree framework (15):

15 $$IG=1-\sum {p(x=k)}^{2}$$

### Implementation of support vector machines

The traditional classifier list employs a SVM to reduce errors and speed up the process^53^. The main benefit of using an SVM is that it performs incredibly well with relatively tiny datasets. Creating a decision border that divides an n-dimensional environment into discrete classes is the ultimate goal of an SVM classifier^54^. The SVM meticulously forms this boundary, known as a hyper plane, using particular points called support vectors. SVM determines the best decision extremity by choosing the border that maximises the degree of separation from the neighbouring data points of all classes. The hyper plane is the best border since it has the largest distance between each class's nearest points. When the data cannot be separated linearly, a revamped form of SVM known as Kernel SVM is used. The data that are not linearly separable are converted into linearly distinct data by Kernel SVM. As a measure of the Support Vector Machine in this study, a sigmoid kernel was used.

### Logistic regression analysis

Logistic regression^55^ was chosen for analysis due to its ease of use and effectiveness during training. Regularisation can be used to reduce overfitting as well. In this instance, the remaining uncorrelated variables can be used to forecast which of two classes the answer variable will belong to. In this procedure, the sigmoid function*σ(t)*, denoted by Eq. (16), is essential:

16 $$\sigma \left(t\right)=\frac{{e}^{t}}{{e}^{t}+1}=\frac{1}{{e}^{-t}+1}$$

The input data are transformed by this S-shaped function onto a scale ranging from 0to 1. Values under 0.5 are categorised as 0, and those greater than 0.5 are classified as 1. The sigmoid function's output also offers the likelihood that the event will occur. For example, if the return value is 0.72, then there is a 72% chance for that event to occur.

## Experimental results and discussions

### Materials

Evaluated our proposed framework on three public benchmark datasets, including ISIC 2019^56^, ISIC 2020^57^and PAD-UFES-20^58^ dataset. These ISIC datasets were taken from the International Symposium of Biomedical Imaging Collaboration (ISBI) for the detection of melanoma. From ISIC 2020, 2700 malignant lesions and 3000 benign samples of 5700 images were utilised in this study. As the mostly recent curated dataset, ISIC 2020 is available at https://challenge2020.isic-archive.com/.

### Results and discussions

**Figure 4:**
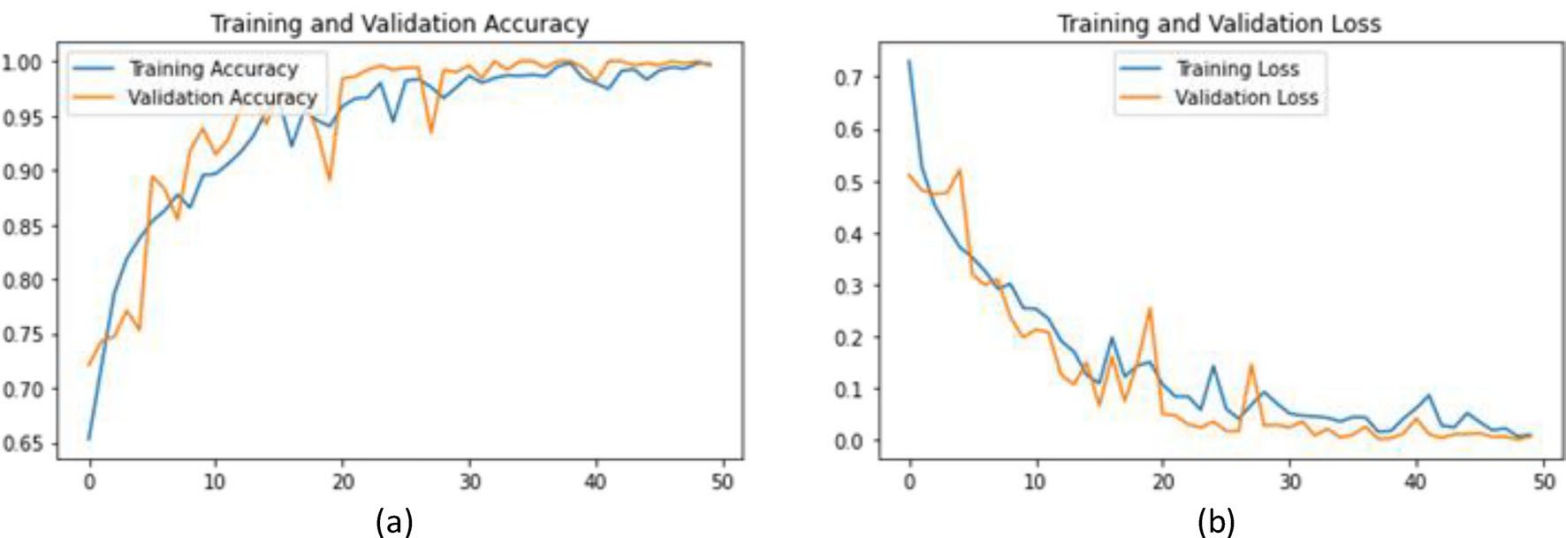
(**a**) Training Accuracy vs Epochs; (**b**) Training Loss vs Epochs.

This section summarises the results of the original CNN model, the creation of a baseline dataset with several categories, and the methods used for preprocessing an image, feature extraction, and classification. The experimental dataset was randomly split 70:30 into training and testing sets, yielding 2302 and 989 samples, respectively. The proposed technique was evaluated in 15 iterations. The training phase was evaluated using the training set for each subsequent run using a tenfold cross-validation approach, with the residual set being used to assess the model's generalisability to new data. The standard CNN model has four convolutional layers, each with 15 filters and a 3*3 pixel size. Binary cross-entropy was chosen as the loss function, while Adam's method was employed as the optimiser. The CNN model was trained with 150 epochs, as shown in Fig. 4. Softmax, the last activation function used, produced the predicted probability. A BSC comprises samples with confidence levels below a predetermined threshold.

Table 4 displays the results of the baseline deck one CNN. Based on the data, CNN had trouble classifying unseen samples, especially when trying to identify cancerous samples.

**Table 4:** Deck one CNN parameters of DDCNN.

Parameters & results	
Epochs	50
Optimizer	Adam
Loss	Binary cross-entropy
Batch size	40
Training accuracy	98.7
Testing accuracy	90.5
Training loss	0.047
Testing loss	1.03

The test set assessed the CNN model and calculated the likelihood of each sample being benign or cancerous. According to Table 5, the model was somewhat over fitted, and changing the hyper parameters or increasing the total count of epochs did not significantly increase accuracy. These results can be ascribed to the CNN model's lightweight and quick nature. After 40 epochs, training for the CNN model stabilised, and no significant changes were seen. The training procedure was therefore stopped after 50 epochs. The difficult samples were divided into a BSC, using this CNN model as the starting point for identifying readily recognisable samples. An alternate classification method was used on the BSC to calculate the difference between an unlabelled sample and a sample labelled benign or malignant.

**Table 5:** BSC dataset sample size.

2 classes	Before aug	After aug
Hairy sample	236	412
Non-hairy sample	417	417
Total	653	829

**Figure 5:**
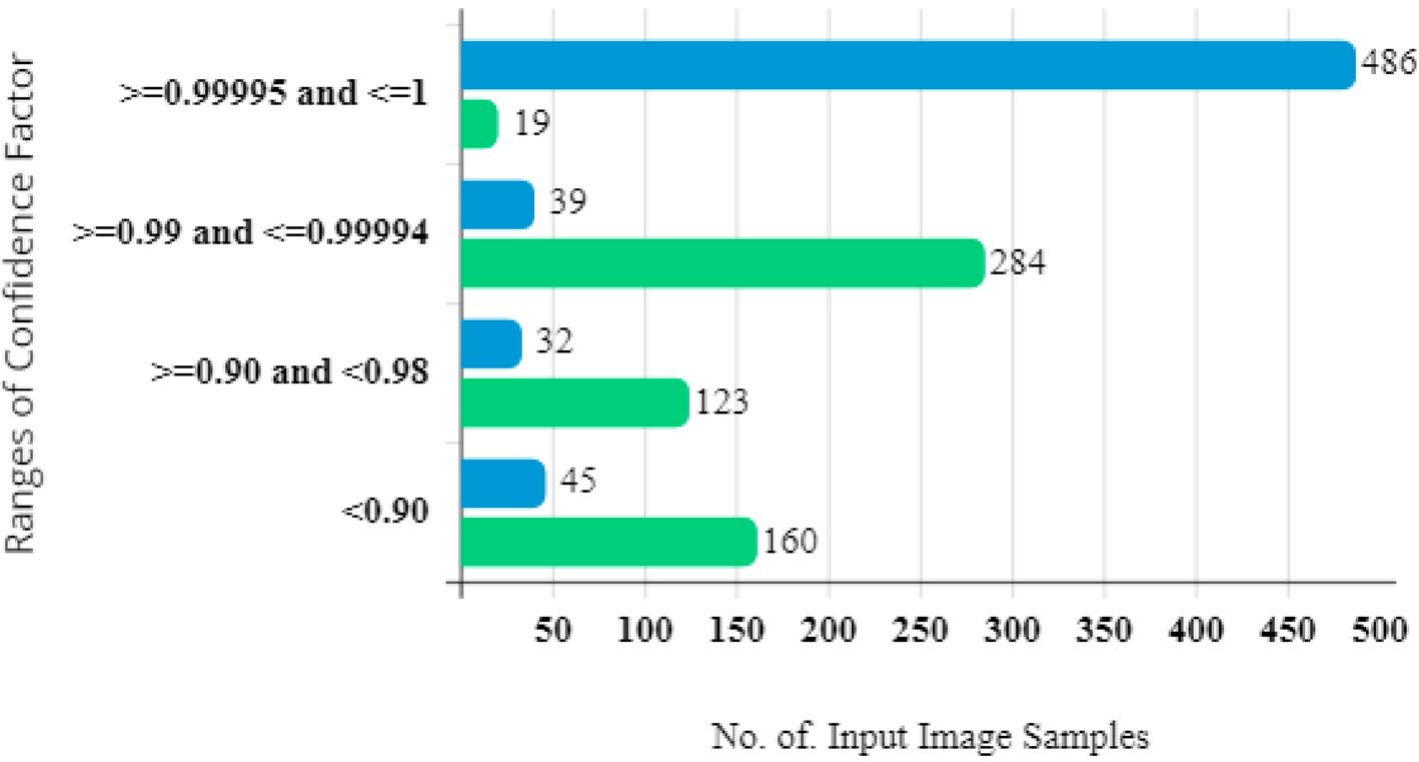
BSC results concerning the confidence factor and number of image samples.

Different ranges of intra-class variance scores were considered using a trial-and-error method. The overall count of samples falling inside each range and the quantity of incorrectly classified samples were calculated. The results in Fig. 5 show a decreasing relationship between the frequency of inaccurate predictions and the intra-class variance score. Samples with an error ratio of more than 30% and confidence factor of less than 0.999995 were moved to a separate dataset. By utilising a different model to train these samples, this phase seeks to increase prediction confidence.

**Figure 6:**
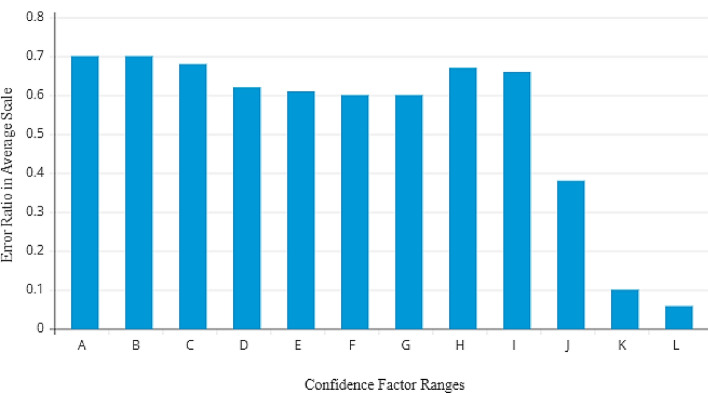
Average error ratio vs. confidence factor ratio.

As seen in Fig. 6, the error ratio increased as the intra-class variance score fell because more samples within that range were incorrectly classified. When the intra-class variance score diminished from 0.999995 to 0.90, the number of incorrectly categorised samples increased from 19 to 92. It is evident that increasing the range and reducing the intra-class variance score caused the error ratio to increase. Notably, 95% of samples with a probability difference greater than 0.999995 were correctly identified. On the other hand, samples with a confidence factor (intra-class variance score) less than 0.999995 were shifted to a newly segregated dataset.

A correlation analysis was performed to thoroughly explore the connection between the confidence factor and error ratio, and the outcomes are displayed in Fig. 6. The confidence factor for each image was determined, and the photos were separated into several ranges represented by the letters *A* to *L*, where *L* refers to the highest confidence factor, and *A* represents the lowest. The decreasing trend seen from Range *A* to *L* shows an inverse link between the confidence factor and error ratio. The error ratio decreases in direct proportion to the confidence factor. By examining several sets, the effect of the confidence component is illustrated. *Set A* displays the largest average error ratio and lowest confidence factor. On the other hand, *Set L* has the lowest average error ratio and the highest confidence factor. Figure 6 reveals that this pattern is consistent over all conceivable ranges and emphasises the negative relationship between the confidence factor and error ratio. With a confidence factor above the cut-off, the baseline CNN was used to classify samples, producing an average error ratio of 0.0437.

Therefore, samples falling within ranges *A* to *J*, as shown in Fig. 6, and having a confidence factor lower than 0.9999965 were moved to the BSC. These samples are considered challenging to categorise because their confidence factor is below the predetermined level, so this segregated dataset is devoted to additional classification efforts.

**Figure 7:**
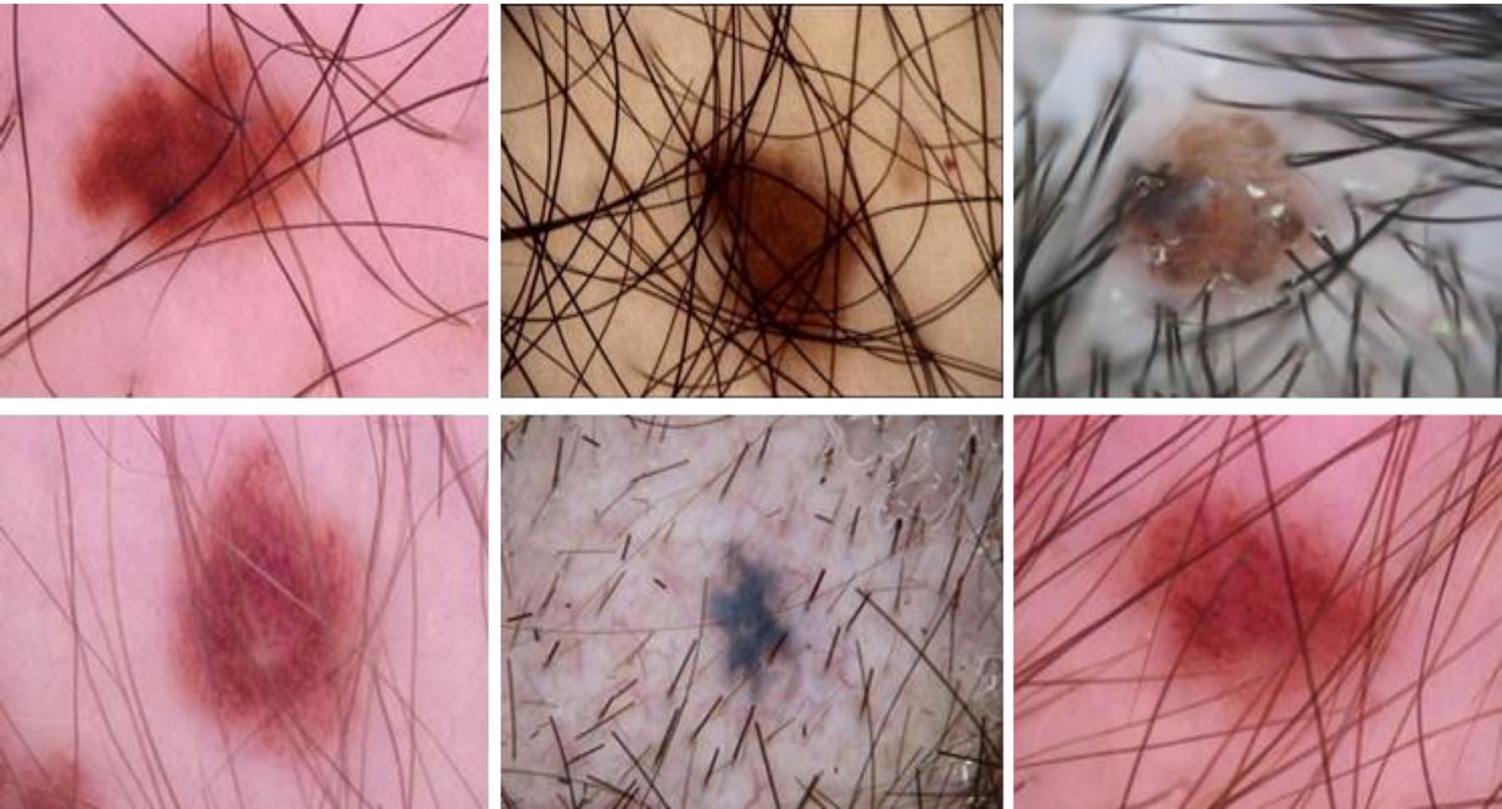
Hairy image samples segregated at the baseline separation channel (BSC).

**Figure 8:**
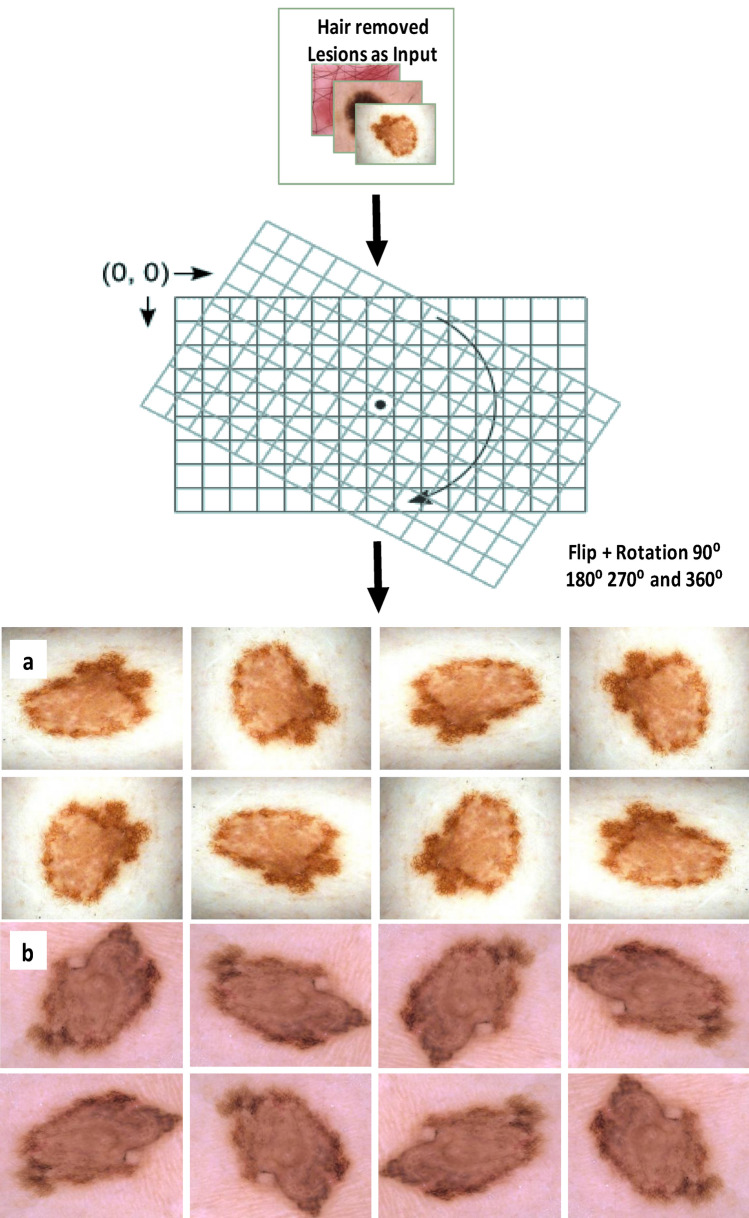
Augmented samples of the skin lesion images.

A glimpse of the samples included in the BSC dataset is shown in Fig. 7. In this BSC phase, hair removal was performed as preprocessing to make extracting characteristics from the samples easier. Figure 8 shows the detailed procedure for hair removal during the no-hair image extraction process. The image was initially converted to grayscale, then a black hat filter was used to emphasise the dark interest areas. The thresholding method was then applied to the filtered image. Last, the inpainting technique removed the hair-containing regions from the original image, leaving behind a hairless image.

Data augmentation is commonly used for benign samples to correct the imbalance in the BSC dataset, where a larger percentage of samples are malignant. Table 5 details the BSC dataset's split and augmentation procedure. This augmentation strategy was performed to reduce overfitting, as seen in the deck one baseline CNN. Overfitting can be successfully reduced using augmentation approaches, and the dataset can be properly balanced.

The augmentation methods used in this work included random rotation to 180,270, and 360, random noise addition, and horizontal flipping. Examples of the augmented data produced using these methods are shown in Fig. 8. The expanded BSC dataset for 150 epochs was used to trained the deck two CNN. Four convolutional layers of 32, 64, 128, and 256 filters, each with a filter size 3*3, make up the CNN architecture. In addition, five dense layers with 256, 128, 64, 32, and 2 units each are included. The third dense layer of the CNN, which recognises and retains 64 crucial features, was chosen to perform feature selection. These desired features were obtained using the ReLU activation function from a fully linked layer with 64 units (features). The CNN predicts whether a lesion is benign or malignant based on these factors or traits. Two units make up the last dense layer, with one unit confirming the presence of cancer. Binary cross entropy was employed as the loss function. Applying this CNN, it was possible to merge the retrieved 64 features with the ABCDE features by saving them as a feature file. Table 8 summarises the acquired results and details the deck two CNN architecture. Although the overfitting was reduced, testing accuracy was still only 85%.

**Figure 9:**
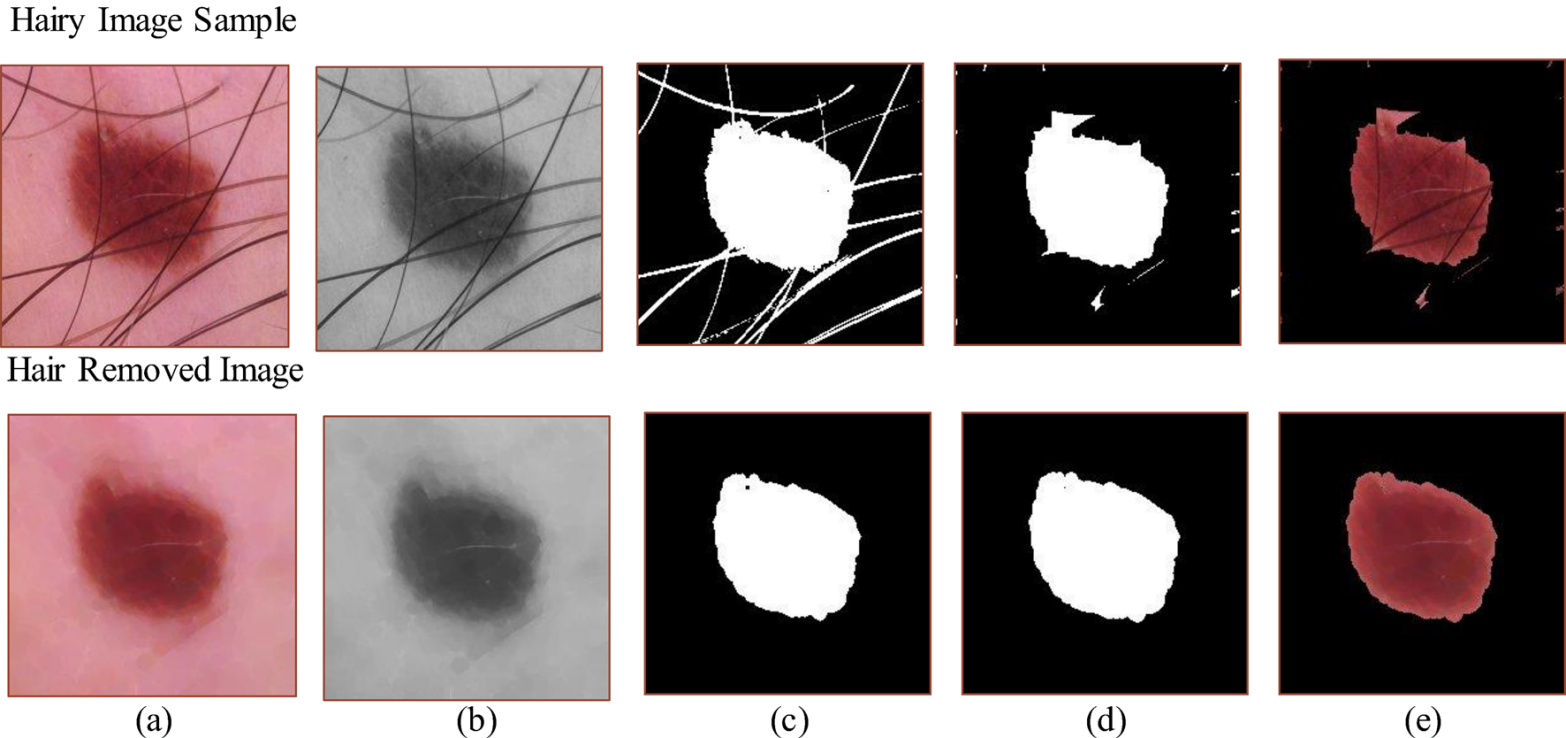
(**a**) Input image; (**b**) Processed scale image; (**c**) Perimeter and feature calculated image; (**d**) Masked image; (**e**) Resulting image.

The separated dataset provides valuable attributes, including asymmetry, border irregularity, colour variegation, and diameter^59^. The image was cropped to highlight the mole and exclude extraneous elements. Only the section that contains the lesion was kept; while everything else was thrown away. This image was further cropped and saved separately. After that, Otsu's threshold was applied to the grayscale image^60^. Following this thresholding process, binary dilation and binary erosion were applied to the result. The final image was saved independently and then masked, which made it possible to extract information like asymmetry, borders, and diameter. The many stages of ABCDE extraction for both hairy and non-hairy images are graphically represented in Fig. 9. This example emphasises the necessity of hair removal to get precise extraction results. It is pertinent to mentioned that extraction will not be successful without effective hair removal.

**Figure 10:**
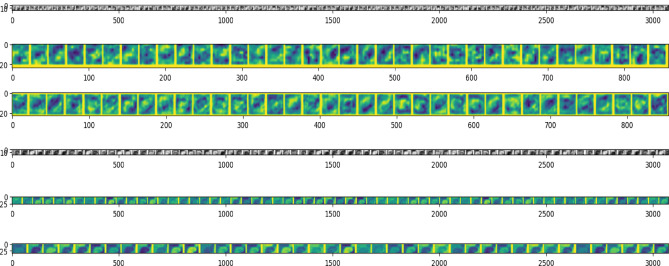
Feature maps of the benign skin lesion input images.

**Figure 11:**
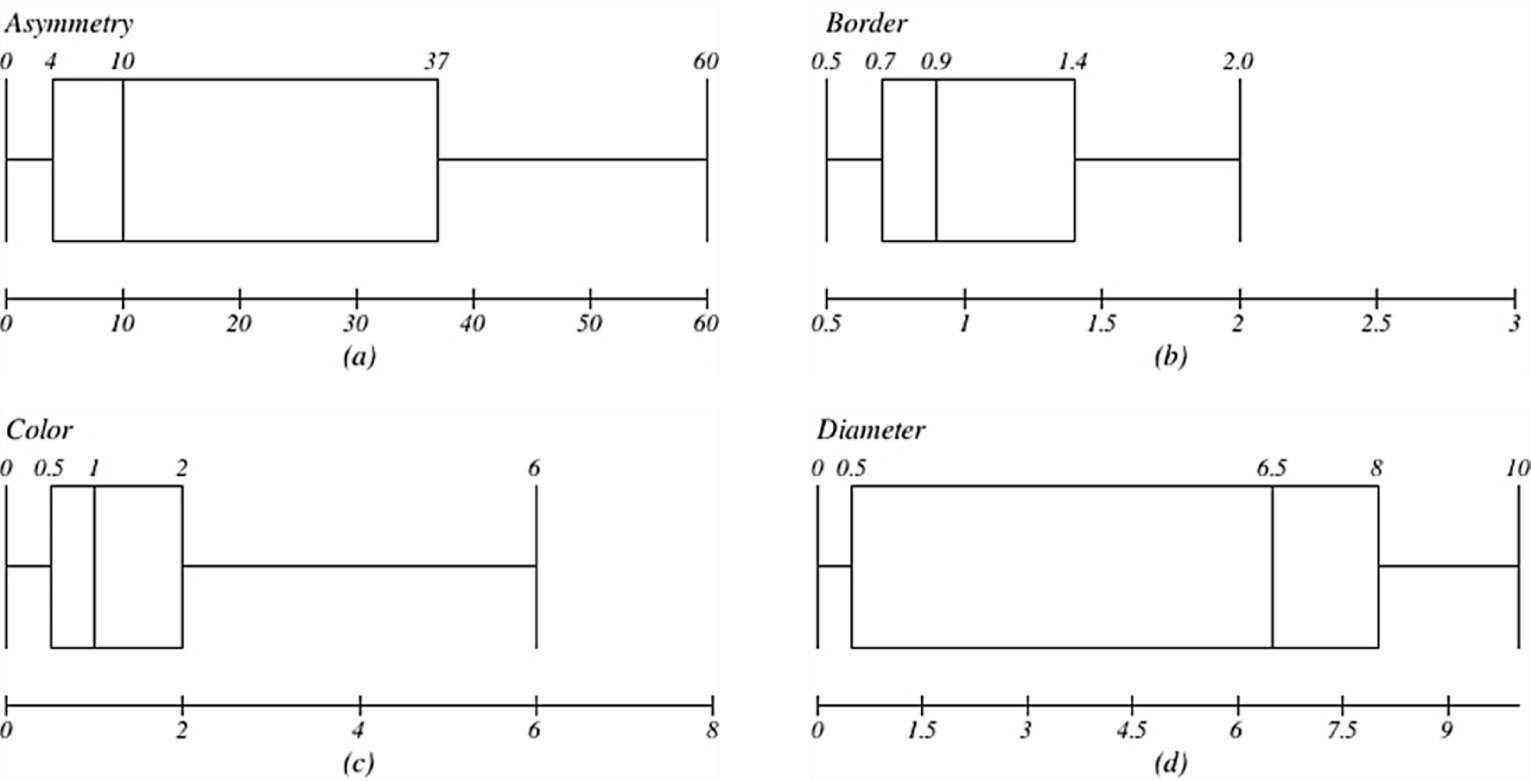
(**a**) Asymmetry, (**b**) border, (**c**) colour, and (**d**) diameter box plots.

Utilising the coloured image is a step in getting the colour characteristic from an image. The RGB data are transformed into HSV numerical values to do this. A visual illustration of how the model learns to recognise various features inside images is provided in Fig. 11, which shows how the CNN extracts features at each layer. The model's internal operations are better understood thanks to this visualisation. It also helps, in some circumstances, in determining the possible reasons why the model fails. It is clear from assessing the feature maps of normal and melanoma images that the lesion's colour and form are essential characteristics. Figure 10 shows a second deck CNN feature map of a benign image obtained in the proposed DDCNN. Unfortunately, there are times when it is difficult to discern between the two groups using these attributes, which reduces accuracy.

The file name is described by four extracted features in each dataset column: asymmetry, border, colour, and diameter. Combining these four features with the 64 features obtained via CNN, 68 features were used in the classification procedure. Seven distinct classifiers were employed to carry out the classification. The results of these classifiers are shown in Table 9. A test size of 30% of the original dataset was employed for each classifier. The model was then utilised to assess untrained images, which acted as the test data when the training phase was complete. Several performance indicators^61^, including accuracy *(Acc)*, precision *(Pre)*, sensitivity *(Sens)*, specificity *(Spec)*, and Area Under Curve (AUC), were calculated to assess and boost the prediction rate. Some performance metrics were used to increase forecast accuracy.

Accuracy, as defined in Eq. (17), quantifies the capacity to distinguish between melanoma and benign instances:

17 $$Acc=\frac{{True}_{Malignant}+{True}_{Benign}}{Total\,number\,of\,input\,skin\,lesion\,images}$$

Precision, defined in Eq. (18), is the ability of the model to correctly classify melanoma:

18 $$Pre=\frac{{True}_{Malignant}}{{True}_{Malignant}+{False}_{Malignant}}$$

Sensitivity, often called recall, measures how well cases of melanoma can be recognised during Eq. (19):

19 $$Sens=\frac{{True}_{Malignant}}{{True}_{Malignant}+{False}_{Benign}}$$

Specificity, expressed in Eq. (20),is a measure of how well benign situations may be recognised:

20 $$Spec=\frac{{True}_{Benign}}{{True}_{Benign}+{False}_{Malignant}}$$

Together, these indicators help to increase the prediction rate and assess the effectiveness of the classification model.

## Discussion

Results indicate that the proposed Double Decker Convolution Neural Network (DDCNN) feature fusion framework was able to predict malignant melanoma while overcoming image problems, such as hair presence, overfitting, time requirements, and low specificity. Nevertheless, it should be noted that this research was limited to binary classification tasks. Future research will examine visual attention approaches to solve more general skin lesion classification issues. A productive methodology will also be used to select features and identify melanoma from a benchmark database. The proposed method is contrasted with current state-of-the-art approaches in Table 6. The suggested technique's high accuracy, AUC, and specificity are particularly noteworthy. In terms of general accuracy, there is still space for growth.

**Table 6:** Parameters and outcome of deck two CNN.

Parameters & results	
Epochs	250
Optimizer	Adam
Loss	Binary cross-entropy
Batch size	16
Training accuracy	95.67
Testing accuracy	92.53
Training loss	0.048
Testing loss	2.03

Tables 7 and 8 overview the outcomes of several classifiers trained with and without the 'F' flag feature. It can be seen that the gradient boosting classifier outperformed the other ensemble classifiers, closely followed by the bagging classifier. The most successful machine learning classifier was determined to be logistic regression.

**Table 7:** Evaluation metrics of different classifiers result for the sample input images without utilising the 'F' flag feature.

Classifiers (Without *'F' Flag Feature*)	Accuracy (%)	Precision (%)	Sensitivity (%)	Specificity (%)
Multi-Layer perceptron^62^	85.47	90.61	81.01	91.89
Gradient boosting classifier^63^	87.6	97.51	88.63	**97.47**
Bagging classifier^64^	88.83	89.6	89.6	90.06
XGBoost classifier^65^	90.87	92.86	85.52	92.41
Decision trees^66^	87.29	91.69	84.32	90.96
Logistic Regression^67^	88.71	91.98	89.45	91.25
SVM^68^	92.28	93.6	90.92	93.67

Significant values are in bold.

**Table 8:** Evaluation metrics of different classifiers for the sample input images trained with the *'*F' flag feature.

Classifiers (With *'F' Flag Feature*)	Accuracy (%)	Precision (%)	Sensitivity (%)	Specificity (%)
Multi-layer perceptron	92.81	89.78	96.69	88.97
Gradient boosting classifier	93.6	98.56	89.67	**98.47**
Bagging classifier	93.75	98.56	88.08	98.4
XGBoost classifier	92.82	94.66	91.12	94.52
Decision trees	87.38	91.69	84.32	90.96
Logistic regression	91.17	91.49	93.35	92.25
SVM	92.77	94.66	89.45	94.38

Significant values are in bold.

To ascertain the efficacy of the 'F' flag feature, it was tested with several ablation trials, as shown in Table 10. The final improved specificity of classifiers confirms the influence of the newly employed biomarker for the early diagnosis of melanoma. It may be optimised to various loss functions and modify hyper-parameters. Gradient boosting classifiers provide a great deal of versatility. Cross-validation was used in this study to test multiple learning rates, and the optimal rate was discovered to be 0.1. Adding the 'F’ flag feature along with Asymmetry, Border, Colour, and Diameter increased the accuracy of the 5 classifiers. The gradient boosting and bagging classifiers had the highest precision, indicating a high percentage of correctly categorised examples, as seen in Table 9. The gradient boosting classifier had the highest specificity, accurately recognising benign cases, while the multi-layer perceptron had the highest recall, detecting the majority of actual cases of melanoma. Different kernels (Polynomial, Gaussian, and Sigmoid) were sought in the support vector machines (SVM) classifier. It was decided to utilise the sigmoid kernel because it produced the best accuracy. Using the ISIC 2020 dataset, the accuracy of the compared different classifiers before the 'F' feature fusion ranged from 87.6% to 92.28%. After adding the 'F' feature fusion, the accuracy varied from 87.38% to 93.75%. The gradient boosting classifier outperformed the others, with the most remarkable specificity of 98.47%.

**Table 9:** The detailed process and time of the proposed framework.

Process	Time taken
Time taken for each epoch in deck 1 CNN	90 s
Total time is taken for first deck CNN	1.2 h
Data preprocessing	0.5 h
Time is taken for epochs in deck 2 CNN	30 s
Total time is taken for second deck CNN	2.5 h
ABCDE an 'F' flag feature fusion and final classification	2.5 h
Total time taken for the entire model execution	7.8 h

The boxplots in Fig. 11 show how ABCDE characteristics are produced. The estimates of the associated attributes, including Asymmetry, Border, Colour, and Diameter, are shown on the x-axis. The ABCD characteristics, where a large number of samples lie above the mean, are notable examples of this transparent discovery. These samples primarily fit into the malignant class, according to further examination. By analysing structures in their values (more or less than the mean), these parameters play a vital role in predicting malignancy and become essential criteria for categorisation. In this instance, the diameter characteristic does not show abundance among samples beyond the mean. However, study shows that larger diameter values are typically linked to malignancy.

The Table 11 demonstrates how various classifiers were evaluated to see which one could correctly categorise melanoma instances. With an accuracy of 93.6%, the gradient boosting classifier fared the best and had the highest success rate compared to the other classifiers.

**Figure 12:**
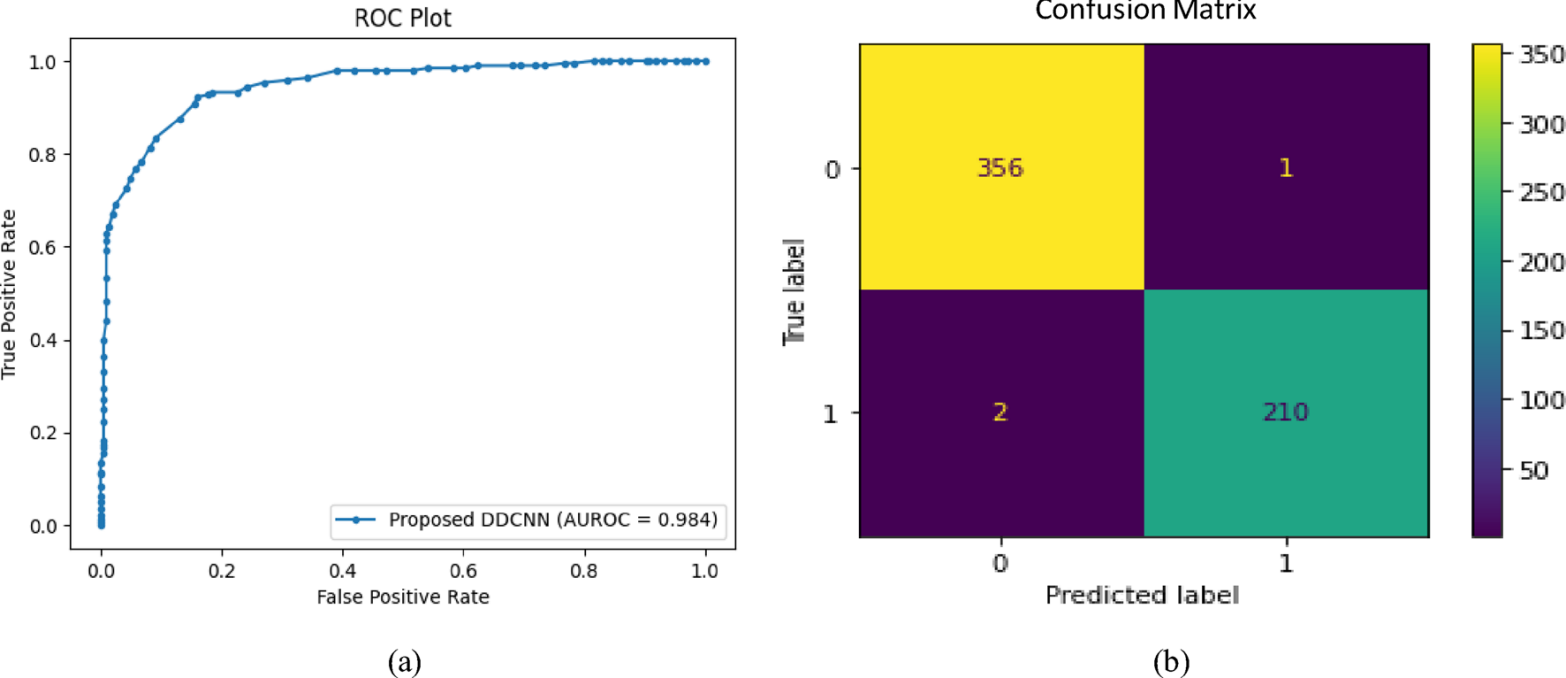
(**a**) ROC plot of the proposed framework; (**b**) Confusion matrix of two classes: benign and malignant.

The sensitivity of a system (how well it can identify positive cases) and specificity (how well it can identify negative cases) are combined in the AUC (Area Under Curve) assessment. This metric aids the comprehension of precision with which a classification system may discriminate between positive and negative classifications. Determining if a system is biased towards positive or negative cases can be challenging when performing individual examination on sensitivity and specificity. Because of this, AUC is regarded as an exemplary metric for evaluating various classification schemes for both positive and negative scenarios. The Receiver Operating Characteristic (ROC) area under the curve is used to compute AUC. This graph displays various sensitivity and specificity combinations as the categorisation threshold varies. The classification approach is closer to a perfect predictor if the AUC value is more prominent. The ROC, which illustrates how well the classification model performs, is shown in Fig. 12a. It has a perfect curve and an extremely effective AUC value of about 0.96. Generated a confusion matrix to see how the model enacts. The amount of true positives, true negatives, false positives, and false negatives predicted by the model is displayed in a grid-like format. The confusion matrix for the test class is shown in Fig. 12b. The model's high specificity is explained by the fact that it correctly predicted the majority of the benign samples, which is helpful. Only five cancerous samples were mistakenly classified as benign.

Table 9 summarises the processing time and temporal performance of the designed system, which was run at 4.20 GHz on an Intel i7-7700 processor with 8 GB of RAM. Despite using two CNN frameworks, the shorter computation time can be attributable to these models' lightweight design. The deck-two CNN needed only 0.5 min for each epoch, compared to the deck-one baseline CNN's average time of 2 min. Techniques for data pre-treatment, such as data augmenting and hair elimination, and were also successfully carried out. The automated and quick ABCDEF classification method worked well. The new 'F' flag feature bio-indicator of melanoma identification clearly improves prediction accuracy. As a result, our model was trained in a mere 10 h, which is exceptionally effective given the size of the dataset, and it took only 1 min to test a batch of samples. Yet, automation of image portions containing hair is one area where personal intervention is still required.

Table 10 reveals the improved specificity of identifying the cancerous lesions from the suspicious benign lesions when the *'*F' feature was incorporated into the training process of all seven classifiers. Among these, the resulting improvement of 7.34% is noted for the best case and 0.49% for the most negligible value. This capability of the proposed DDCNN ‘F’ flag feature fusion confirms its ability to classify melanoma, which is critical for early diagnosis.

**Table 10:** Improvement of specificity of techniques using the 'F' flag feature.

Classifiers	Improved specificity in %
Decision trees	+ 0.09
SVM	+ 0.49
XGBoost classifier	+ 1.95
Logistic regression	+ 2.46
Bagging classifier	+ 4.92
Gradient boosting classifier	+ 6.00
Multi-layer perceptron	+ 7.34

The classification accuracy (%) of the proposed DDCNN 'F' flag feature fusion framework for the ISIC 2020 dataset was determined to be 93.75%, as shown in Table 11. Comparatively, the classification accuracy (%) of other state-of-the-art methods^69–75^ ranged from 81.8 to 92.4. Moreover, the proposed framework achieved the highest specificity (98.4%) and precision rate (98.56%) compared to other cutting-edge classifiers.

**Table 11:** A qualitative assessment of the feature fusion framework is conducted compared with several cutting-edge techniques.

Feature fusion frameworks	Accuracy (%)	Sensitivity (%)	Specificity (%)	Precision (%)
Abayomi-Alli et al.^69^	92.18	80.77	95.1	–
Almaraz-Damian et al.^70^	92.4	86.41	90	92.08
Guo et al.^71^	86.6	55.6	78.5	–
Jane et al.^72^	92.15	86.48	96.8	96.96
Mukherjee et al.^73^	87.2	87.4	86.8	–
Mukherjee et al.^74^	85.9	86.2	85.5	–
Singh et al.^75^	81.8	70	82.6	–
Proposed work DDCNN	93.75	88.08	98.4	98.56

### Significance and limitations

The ‘F’ flag feature contributes as an influencing morphological feature that helps in the classification of melanoma at early stages. Also, the proposed DDCNN-F handles the images occlusion with hair artefacts separately from BSC. The fusion of the feature with each pretrained model confirms the improvise in the accuracy and specificity. This will greatly facilitate the automated detection of melanoma in early stages which benefits the patients and prevent severe metastasis. Though the proposed model achieves better specificity and precision, it still finds some difficulties for the over-hairy images, which are not effectively handled by our hair removal algorithm. Due to higher order inpainting, the images may have leaks in certain pigment features of the dermoscopic image. This should be taken care of so that the loss of 6.25% can still be reduced and that would result in better accuracy.

## Conclusion and future work

This study proposes a framework composed of a double-decker convolution neural network (DDCNN) with the ‘F’ flag feature indicator to categorise dermoscopy images as benign nevus or malignant melanoma. The method employs a base CNN to categorise smaller data. Herein, an apportioned dataset for difficult samples was produced based on the intra-class variance score. In this dataset, hair removal was done to simplify the categorisation of samples. Rotation, flipping, and random noise addition were used as data augmentation techniques to balance the dataset and improve the proportion of benign samples. The samples were subsequently trained using a different CNN model to extract bottleneck CNN characteristics. The photos also contained ABCDEF features, which were all retrieved and merged. The influencing 'F' flag feature incorporated in the second deck of the proposed framework plays a vital role in identifying melanoma samples from regular lesions. This 'F' feature, fused with other bio-indicators of melanoma, showed to outperform other detection methods in terms of accuracy. The retrieved features were used to classify the images using a combination of machine learning, deep learning, and ensemble classifiers, and then the accuracy of each method was compared. For samples with easy identification, the first module's accuracy in the initial categorisation stage was 95%. A deep learning classifier (Multi-Layer Perceptron) obtained 91.21% accuracy in the second stage using the segregated dataset. In comparison, the machine learning classifier (Logistic Regression) achieved an accuracy rate of 91%, and the ensemble of classifiers (Gradient Boosting) achieved 92% accuracy. Combining the ABCDEF features significantly increased the proposed DDCNN ‘F’ feature fusion algorithm’s overall performance in melanoma classifications, resulting in 93.75% accuracy under the extensive evaluations. Our future research will be intended to develop a web application with automated reports on ABCDEF features value with confidence factor of being melanoma, that helps in the early diagnosis of skin cancer extended towards other medical conditions with trending feature fusion on multimodal images^76^. Moreover, the extension of this model can be rigorously fine-tuned for other non-melanoma skin disease diagnosis and can further implemented with staging with treatment planning.

## Data Availability

The ISIC 2019 and ISIC 2020 data are available at the following Kaggle link: https://challenge2020.isic-archive.com. The proposed feature fusion framework code on DDCNN-F can be shared upon reasonable request to the corresponding author, premaladha@ict.sastra.edu.
